# Plenary Abstracts

**DOI:** 10.1080/24740527.2021.1914206

**Published:** 2021-04-22

We have an increasingly robust armoury as to how to stratify neuropathic pain patients according to symptoms, sensory testing and more advanced techniques such as neurophysiology, genetics and functional imaging. These are now being applied at scale both in large collaborative research consortia and in some cases within national health services. In parallel to these technological advances harmonised data collection and storage is enabling advanced multi-modal data analysis and correlation with long term health outcomes. The application of these techniques is helping us to: identify conditions which were not previously understood to have a neuropathic component, identify those individuals at highest risk of neuropathic pain and stratify patients living with neuropathic pain in a clinically meaningful way. I will discuss how these approaches are enhancing our understanding of neuropathic pain with the ultimate goal of not only developing novel treatment strategies but also better targeting of existing treatments to those most likely to respond.

**Learning objectives:** At the end of this lecture I hope that participants will understand:

- The diagnostic approach to neuropathic pain ranging from screening tools to advanced technologies.
- How some of the newer techniques such as sensory profiling and genomics may help us stratify neuropathic pain patients.
- New approaches to treatment of neuropathic pain both in terms of efforts to develop novel treatments but also better targeting of the existing treatments through patient stratification.

