# Supplementary material for: Plenary Abstracts
Source: Can J Pain. 2021 Apr 22;5(2):A1–2. doi: 10.1080/24740527.2021.1914206 (PMC8210882; doi:10.1080/24740527.2021.1914206)
Supplement: Related texts

# Abstract: The spinal circuits of itch and pain

**Authors:** Sarah Ross

The dorsal horn of the spinal cord is the first node at which sensory input is integrated within neural networks. However, our understanding of how these neural networks transform information remains limited. Here, we use a combination of genetic manipulation and 2P Ca2+ imaging to gain insight into this circuitry. In the first study, we examine the neural basis of morphine-induced itch. We find that spinal dynorphin (Pdyn) neurons are both necessary and sufficient for morphine-induced itch in mice. Moreover, agonism of the kappa-opioid receptor alleviated morphine-induced itch in mice and nonhuman primates. Thus, our findings reveal that spinal morphine causes itch through a mechanism of disinhibition. In the second study, we examine the neural basis of capsaicin-induced allodynia at a population level. We find that intradermal capsaicin triggers activity in a subset of excitatory neurons in the dorsal horn that persists for approximately five minutes. Thereafter, a larger population of neurons show emergent activity in response to low threshold input, consistent with the perceptual experience of allydynia. Finally, we use an approach called Cell-type Identification by Ca2+-coupled Activity though Drug Activation (CICADA) classify excitatory cell types in order to analyze which populations of excitatory neurons are involved in which aspects of spinal processing. Finally, we speculate about the channels through which noxious and pruritic information are conveyed to the brain.

**Learning Objectives:** Understand how spinal morphine causes itch and why kappa opioid receptor agonists reverse this side-effect.

Visualize the neural manifestation of allodynia and understand which spinal populations are involved.

Consider (reconsider?) how noxious information is conveyed to the brain

# Abstract: Learning Health Systems for Optimized Care and Real-World Research Discovery and Innovation

**Authors:** Sean Mackey

The United States National Academy of Medicine (NAM) called for the development of national patient registries and Learning Healthcare Systems (LHS). This call has been echoed by other countries. I will describe the rationale and the power of patient registries and LHSs. As envisioned, LHS leverages an integrated digital infrastructure to provide data-based and coordinated care that is available just-in-time to the clinician and that is centered on the patient. Additionally, there is increasing interest in the use of real world data/real world evidence (RWD/RWE) to represent a much broader and more diverse patient experience compared to the traditional RCTs. Further, expansion of RWD/RWE allows for very large sample sizes that promote the detection of infrequent events, treatment-treatment interactions, and better account for heterogeneity of treatment effects. LHSs are critical to advancing RWD/RWE to inform patient care and to advanced more generalizable knowledge.

In this session, I will survey the field of LHS platforms, and illustrate its power using the Collaborative Health Outcome Information Registry (CHOIR) as a use case. CHOIR is an open source and free platform created at Stanford, available to academic institutions. Key topics include 1) clinical decision support features of CHOIR, 2) obtaining research-grade clinical data as a part of routine clinical care, 3) using LHS for clinical trials and rapid piloting of clinical interventions, 4) real-time aggregation and summarization of LHS data to provide on-going decision support in the perioperative and outpatient environments, and 5) research efforts and publications made possible by large-scale LHS platforms like CHOIR

**Learning Objectives:** Identify defining features of patient registries and learning healthcare systems (LHS), and recognize the roles LHS platforms play in acute and chronic pain management.

Evaluate the impact of LHS platforms on patient care and research activities.

Delineate the aspects of registries and LHS and appreciate the data and process utility of them, both as a tangible IT infrastructure and as a profound cultural change in care delivery.

Describe features of model open source LHS platforms and how to implement them in their home institution.

Describe results from both a chronic and perioperative registry identifying factors of high risk and good pain care
